# Supplementary material for: Did socioeconomic inequalities in overweight and obesity in South African women of childbearing age improve between 1998 and 2016? A decomposition analysis
Source: PLOS Glob Public Health. 2024 Nov 14;4(11):e0003719. doi: 10.1371/journal.pgph.0003719 (PMC11563443; doi:10.1371/journal.pgph.0003719)
Supplement: S1 Table — (DOCX) [file pgph.0003719.s002.docx]

**S1 Table. Decomposition of the concentration index for overweight among women of childbearing age 15 – 49 years, South Africa, 1998 and 2016**

|  | **1998** | | |  | **2016** | | |
| --- | --- | --- | --- | --- | --- | --- | --- |
|  | **Concentration index** | **Elasticity** | **Contribution** |  | **Concentration index** | **Elasticity** | **Contribution** |
| Age, years | 0.018***  (0.003) | 0.965***  (0.061) | 0.018***  (0.003) |  | 0.015***  (0.004) | 0.771***  (0.056) | 0.011***  (0.003) |
| Socioeconomic status | 0.274***  (0.003) | 0.080  (0.052) | 0.022  (0.014) |  | 0.015***  (0.004) | 0.180***  (0.059) | 0.040***  (0.013) |
| **Race** | | | | | | | |
| Black African | -0.175***  (0.007) | 0.242***  (0.063) | -0.042***  (0.011) |  | 0.220***  (0.005) | 0.273**  (0.123) | -0.016**  (0.008) |
| Coloured | 0.378***  (0.020) | 0.015*  (0.009) | 0.006  (0.003) |  | -0.060***  (0.033) | 0.009  (0.008) | 0.004  (0.003) |
| Asian/Indian | 0.667***  (0.018) | -0.003  (0.004) | -0.002  (0.003) |  | 0.405***  (0.030) | 0.003  (0.003) | 0.002  (0.002) |
| White |  |  |  |  |  |  |  |
| **Education** | | | | | | | |
| No schooling | -0.256***  (0.012) | 0.010  (0.022) | -0.003  (0.006) |  | 0.817***  (0.032) | 0.026*  (0.015) | -0.010*  (0.006) |
| Primary | 0.154***  (0.009) | 0.018  (0.036) | 0.003  (0.006) |  | -0.392  (0.008) | 0.255**  (0.108) | -0.002  (0.002) |
| Secondary | 0.520***  (0.024) | -0.015**  (0.007) | -0.008**  (0.003) |  | -0.008***  (0.030) | 0.026*  (0.015) | 0.014*  (0.008) |
| Tertiary |  |  |  |  |  |  |  |
| **Employment status** | | | | | | | |
| Employed | 0.230***  (0.012) | 0.043***  (0.013) | 0.010***  (0.003) |  | 0.541***  (0.018) | 0.016  (0.012) | 0.002  (0.002) |
| **Marital status** | | | | | | | |
| Married/living together | 0.027**  (0.012) | 0.062*  (0.032) | 0.002  (0.001) |  | 0.152**  (0.020) | 0.012  (0.026) | 0.001  (0.001) |
| Single/never married | -0.033***  (0.009) | -0.014  (0.044) | 0.000  (0.002) |  | 0.043**  (0.011) | -0.074  (0.051) | 0.002  (0.001) |
| Widowed or divorced |  |  |  |  |  |  |  |
| **Area of residence** | | | | | | | |
| Urban | 0.280***  (0.008) | 0.061**  (0.026) | 0.017**  (0.007) |  | -0.023***  (0.010) | -0.034  (0.025) | -0.007  (0.005) |
| **Lifestyle** |  |  |  |  |  |  |  |
| Smoking | 0.244***  (0.026) | -0.028***  (0.007) | -0.007***  (0.002) |  | 0.213***  (0.051) | -0.002  (0.004) | 0.000  (0.001) |
| Residual |  |  | <0.000  (0.009) |  |  |  | -0.001  (0.010) |
| **Total** |  |  | 0.016  (0.011) |  |  |  | 0.038***  (0.010) |

Significance levels are denoted as follows: *** p< 0.01, ** p< 0.05, *p< 0.10. Bootstrapped standard errors are displayed in parentheses
