# Supplementary material for: Did socioeconomic inequalities in overweight and obesity in South African women of childbearing age improve between 1998 and 2016? A decomposition analysis
Source: PLOS Glob Public Health. 2024 Nov 14;4(11):e0003719. doi: 10.1371/journal.pgph.0003719 (PMC11563443; doi:10.1371/journal.pgph.0003719)
Supplement: S2 Table — (DOCX) [file pgph.0003719.s003.docx]

**S2 Table. Decomposition of the concentration index for obesity among women of childbearing age (15 – 49 years), South Africa, 1998 and 2016**

|  | **1998** | | |  | **2016** | | |
| --- | --- | --- | --- | --- | --- | --- | --- |
|  | **Concentration index** | **Elasticity** | **Contribution** |  | **Concentration index** | **Elasticity** | **Contribution** |
| Age, years | 0.018***  (0.003) | 1.449***  (0.121) | 0.026***  (0.005) |  | 0.015***  (0.004) | 1.260***  (0.104) | 0.018***  (0.006) |
| Socioeconomic status | 0.274***  (0.003) | 0.280***  (0.103) | 0.077***  (0.028) |  | 0.015***  (0.004) | 0.415***  (0.097) | 0.091***  (0.021) |
| **Race** | | | | | | | |
| Black African | -0.175***  (0.007) | 0.477***  (0.116) | -0.083***  (0.020) |  | 0.220***  (0.005) | 0.616***  (0.226) | -0.037***  (0.014) |
| Coloured | 0.378***  (0.020) | 0.031**  (0.015) | 0.012**  (0.006) |  | -0.060***  (0.033) | 0.027*  (0.016) | 0.011*  (0.006) |
| Asian/Indian | 0.667***  (0.018) | 0.002  (0.008) | 0.001  (0.005) |  | 0.405***  (0.030) | 0.006  (0.005) | 0.005  (0.004) |
| White | - | - | - |  | - | - | - |
| **Education** | | | | | | | |
| No schooling | - | - | - |  | - | - | - |
| Primary | -0.256***  (0.012) | 0.048  (0.046) | -0.012  (0.012) |  | 0.817***  (0.032) | 0.014  (0.024) | -0.006  (0.010) |
| Secondary | 0.154***  (0.009) | 0.047  (0.074) | 0.007  (0.011) |  | -0.392  (0.008) | 0.201  (0.176) | -0.002  (0.003) |
| Tertiary | 0.520***  (0.024) | -0.016  (0.013) | -0.008  (0.007) |  | -0.008***  (0.030) | 0.018  (0.026) | 0.010  (0.014) |
| **Employment status** | | | | | | | |
| Employed | 0.230***  (0.012) | 0.039  (0.026) | 0.009  (0.006) |  | 0.541***  (0.018) | 0.008  (0.023) | 0.001  (0.004) |
| **Marital status** | | | | | | | |
| Married/living together | 0.027**  (0.012) | 0.102  (0.070) | 0.003  (0.002) |  | 0.152**  (0.020) | -0.048  (0.062) | -0.002  (0.003) |
| Single/never married | -0.033***  (0.009) | -0.021  (0.092) | 0.001  (0.003) |  | 0.043**  (0.011) | -0.225*  (0.118) | 0.005  (0.004) |
| Widowed or divorced | - | - | - |  | - | - | - |
| **Area of residence** | | | | | | | |
| Urban | 0.280***  (0.008) | 0.125**  (0.050) | 0.035**  (0.014) |  | -0.023***  (0.010) | -0.068  (0.042) | -0.015  (0.009) |
| **Lifestyle** | | | | | | | |
| Smoking | 0.244***  (0.026) | -0.034***  (0.013) | -0.008**  (0.003) |  | 0.213***  (0.051) | 0.000  (0.007) | 0.000  (0.002) |
| Residual |  |  | <0.000  (0.017) |  |  |  | -0.001  (0.018) |
| **Total** |  |  | 0.058***  (0.018) |  |  |  | 0.080***  (0.019) |

Significance levels are denoted as follows: *** p< 0.01, ** p< 0.05, *p< 0.10. Bootstrapped standard errors are displayed in parentheses
