# Supplementary material for: Did socioeconomic inequalities in overweight and obesity in South African women of childbearing age improve between 1998 and 2016? A decomposition analysis
Source: PLOS Glob Public Health. 2024 Nov 14;4(11):e0003719. doi: 10.1371/journal.pgph.0003719 (PMC11563443; doi:10.1371/journal.pgph.0003719)
Supplement: S3 Table — (DOCX) [file pgph.0003719.s004.docx]

**S3 Table: Decomposition of change in concentration index for overweight and obesity among women of childbearing age (15 – 49 years), South Africa, 1998 – 2016**

|  | **Obesity** | | |  | **Overweight** | | |
| --- | --- | --- | --- | --- | --- | --- | --- |
|  | **Rural** | **Urban** | **Total** |  | **Rural** | **Urban** | **Total** |
| Age, years | 0.011  (0.011) | -0.005  (0.010) | -0.008  (0.008) |  | 0.007  (0.007) | -0.004  (0.006) | -0.006  (0.005) |
| Socioeconomic status | 0.039  (0.042) | 0.021  (0.037) | 0.015  (0.035) |  | 0.019  (0.022) | 0.020  (0.021) | 0.018  (0.019) |
| **Population group** | | | | | | | |
| Black African | 0.023  (0.014) | 0.040***  (0.015) | 0.046*  (0.024) |  | 0.015**  (0.006) | 0.024***  (0.009) | 0.026**  (0.013) |
| Coloured | - | - | -0.001  (0.008) |  | - | - | -0.002  (0.005) |
| Asian/Indian | - | - | 0.004  (0.007) |  | - | - | 0.004  (0.004) |
| White | - | - | - |  | - | - | - |
| **Education** | | | | | | | |
| No schooling | - | - | - |  | - | - | - |
| Primary | 0.005  (0.015) | 0.004  (0.024) | 0.007  (0.015) |  | 0.000  (0.007) | -0.017  (0.012) | -0.008  (0.008) |
| Secondary | -0.005  (0.016) | -0.016  (0.019) | -0.009  (0.012) |  | 0.002  (0.007) | -0.011  (0.010) | -0.005  (0.006) |
| Tertiary | -0.004  (0.011) | 0.035  (0.029) | 0.018  (0.015) |  | 0.008  (0.006) | 0.035*  (0.018) | 0.022**  (0.009) |
| **Employment status** | | | | | | | |
| Employed | -0.007  (0.008) | -0.005  (0.007) | -0.008  (0.007) |  | -0.004  (0.004) | -0.007*  (0.004) | -0.007**  (0.004) |
| **Marital status** | | | | | | | |
| Married/living together | 0.003  (0.007) | -0.014*  (0.008) | -0.005  (0.004) |  | 0.002  (0.003) | -0.007*  (0.004) | -0.001  (0.002) |
| Single/never married | 0.000  (0.007) | 0.007  (0.009) | 0.004  (0.005) |  | 0.001  (0.003) | 0.002  (0.004) | 0.001  (0.002) |
| Widowed or divorced | - | - | - |  | - | - | - |
| **Area of residence** | | | | | | | |
| Urban | - | - | -0.049***  (0.017) |  | - | - | -0.024***  (0.009) |
| **Lifestyle** | | | | | | | |
| Smoking | 0.005  (0.007) | 0.004*  (0.002) | 0.008**  (0.004) |  | 0.008**  (0.003) | 0.002*  (0.001) | 0.006***  (0.002) |
| Residual | 0.012  (0.043) | 0.004  (0.033) | 0.000  (0.025) |  | 0.013  (0.021) | -0.001  (0.019) | -0.001  (0.014) |
| **Total** | **0.083*****  **(0.000)** | **0.075*****  **(0.000)** | **0.023*****  **(0.003)** |  | **0.070*****  **(0.000)** | **0.037*****  **(0.000)** | **0.022*****  **(0.000)** |

Significance levels are denoted as follows: *** p< 0.01, ** p< 0.05. Bootstrapped standard errors are displayed in parentheses.
