# Supplementary material for: Did socioeconomic inequalities in overweight and obesity in South African women of childbearing age improve between 1998 and 2016? A decomposition analysis
Source: PLOS Glob Public Health. 2024 Nov 14;4(11):e0003719. doi: 10.1371/journal.pgph.0003719 (PMC11563443; doi:10.1371/journal.pgph.0003719)
Supplement: Attachment — Submitted filename: Reviewers comments.pdf [file pgph.0003719.s006.pdf]

Did socioeconomic inequalities in overweight and obesity in South African women of childbearing age improve between 1998 and 2016? A decomposition analysis

This is a well written article. I just have few comments that will make the article clearer to the readers:

1. Concentration Index Adjustment: The concentration index (CI) is a widely utilized measure in socioeconomic inequality analysis. However, it is crucial to address whether the CI has been adjusted to account for any biases or variations that could affect the results. To ensure clarity, the authors should include a detailed statement in the methodology section, specifying if the CI was adjusted. If adjustments were made, the authors should explain the rationale and the methods used to adjust the CI. Conversely, if no adjustment was made, the authors should provide a clear explanation for this decision

2. The Oaxaca Decomposition Approach: The article currently presents the use of the Oaxaca decomposition approach, but the rationale behind its selection remains unclear. To enhance the readers' comprehension, the authors should elaborate on why this specific method was chosen. They should provide a brief explanation of the Oaxaca decomposition approach, highlighting its relevance and advantages in the context of their study. This expanded explanation in the methodology section will offer a thorough understanding of the approach and its application in analyzing the data.
