# Supplementary material for: Did socioeconomic inequalities in overweight and obesity in South African women of childbearing age improve between 1998 and 2016? A decomposition analysis
Source: PLOS Glob Public Health. 2024 Nov 14;4(11):e0003719. doi: 10.1371/journal.pgph.0003719 (PMC11563443; doi:10.1371/journal.pgph.0003719)
Supplement: Attachment — Submitted filename: Resubmission letter 20240505.docx [file pgph.0003719.s005.docx]

4 May 2024

The Editor,

PLOS Global Public Health

**Did socioeconomic inequalities in overweight and obesity in South African women of childbearing age improve between 1998 and 2016? A decomposition analysis**

Dear Editor,

Thank you for your feedback. We have addressed the issues highlighted in italics and responded in bold (blue font).

*‘1. Please ensure that the Author List in your manuscript file matches the Author List in the online submission form, including initials and order. Currently, author John Ele-Ojo Ataguba is listed in the online submission form but John E. Ataguba from the manuscript.*

**The author list in the manuscript file matches the author list in the online submission form, including initials and order. Author John E. Ataguba is listed in the online submission from and is also John E. Ataguba from the manuscript file**

*2. Your study includes child participants, please update your Ethics Statement with confirmation that written informed consent was obtained from the parent/guardian of each participant under 18 years of age.*

**This paper uses publicly available data that have received ethics approval and informed consent was sought before conducting the Demographic and Health Survey. While this is the case, we still obtained ethics approval from the University of Cape Town’s Human Research Ethics Committee. Further, we did not need to obtain our own individual consent from parents/guardians as this was based on the publicly available Demographic and Health Survey.**

We have resolved the issues and resubmit the paper for your consideration to publish in the PLOS Global Public Health journal.

Yours sincerely,

Mweete Nglazi, on behalf of all co-autho
