# Supplementary material for: Did socioeconomic inequalities in overweight and obesity in South African women of childbearing age improve between 1998 and 2016? A decomposition analysis
Source: PLOS Glob Public Health. 2024 Nov 14;4(11):e0003719. doi: 10.1371/journal.pgph.0003719 (PMC11563443; doi:10.1371/journal.pgph.0003719)
Supplement: Attachment — Submitted filename: Response to Reviewers.docx [file pgph.0003719.s007.docx]

PGPH-D-24-00977

Did socioeconomic inequalities in overweight and obesity in South African women of childbearing age improve between 1998 and 2016? A decomposition analysis

PLOS Global Public Health

Dear Editor,

We would like to thank the reviewers for their helpful comments to strengthen the above captioned manuscript. Please find below a point-by-point response to the reviewers comments.

Journal Requirements:

Additional Editor Comments (if provided):

1. The manuscript would benefit from a more detailed explanation of how the concentration index was adjusted for relevant variables. Additionally, it is essential to provide a clearer rationale for choosing the Oaxaca Decomposition Approach in the context of this study. Please elaborate on these points to enhance the methodological transparency and rigor.

**Response**: Thanks for reading through our manuscript and for your comments. This comment was also picked up by reviewer #2. We have expanded that sub-section to explain the issue of adjustments.

Please see the following in addition to other edits to address this comment.

“It has been suggested to normalise the concentration index when the variable of interest is binary like “obesity” and “overweight” as used in this paper to ensure that the theoretical value of C remains in the [-1,1] range [46]. However, the approaches suggested in the literature [46, 47] for normalising and adjusting the concentration index may produce results counterintuitive for policy to reduce the burden of overweight and obesity among the most affected populations [48]. Therefore, this paper did not normalise the concentration index but presents the results for the standard concentration index [48]. Further, the concentration index may be standardised to account for age/sex variations or for key explanatory variables [34, 45]. This adjustment was not necessary in this paper as the decomposition analysis that follows using these variables is a detailed approach to assessing and explaining key factors contributing to the concentration index.”

“Three decomposition approaches may be used, namely the Oaxaca-type method, the total differential approach [49] and a recent method proposed by Ataguba and colleagues [50] that focuses on inequalities between- and within-socioeconomic groups. The Oaxaca-type decomposition approach used in this paper decomposes the change in socioeconomic inequality in overweight and obesity over time (i.e., between two time periods denoted by subscripts t and t-1) into changes in the concentration index (C_kt and C_(kt-1)) and elasticities (η_kt and η_(kt-1)) of the factors explaining and contributing to changes in socioeconomic inequalities in overweight and obesity. For each contributing factor or variable, changes in the elasticities between two time periods are weighted by the concentration index and the changes in the concentration index by the elasticities, as shown in equation 4. One limitation of the Oaxaca-type method was the difficulty disentangling changes within elasticities [49], which the total differential approach (TDA) presents as an alternative. Because this paper was not intended to unpack changes within elasticities, which is of limited policy relevance, it uses the Oaxaca-type decomposition approach widely used in applied research. Further, the total differential approach is only applicable for small changes in inequality [49], which makes its application rather restrictive compared to the Oaxaca-type approach that is encompassing. Also, the Oaxaca-type decomposition has the added advantage of being easily understood. The recently proposed decomposition framework by Ataguba and colleagues [50] decomposes changes in socioeconomic inequalities into changes between- and within-socioeconomic group health inequalities. It does not show variables or factors that explain changes in socioeconomic inequalities, a major focus of this paper.”

2. To improve clarity and replicability, we recommend including a flowchart detailing the process of assembling the final sample. Furthermore, please provide explicit information regarding the inclusion and exclusion criteria used for the analysis. These additions will enhance the comprehensibility of the methodology section.

**Response**: A flowchart has been added detailing the process used to assemble the data, especially how the final sample was arrived at. Please see “Figure XXX”

Additionally, information on the inclusion and exclusion criteria for the analysis, we have noted the major reasons for the choice of the Oaxaca decomposition approach for analysis as opposed to the total differential approach or the between- and within-SES group approach. One major reason was that the TDA is only valid for small changes in inequality and not a n encompassing approach. Regarding the choice of variables, the following is contained in the revised manuscript:

“…. Variable selection was based on the Dahlgren and Whitehead model [39] concerning important social determinants of obesity and overweight, and availability across the SADHS datasets.”

3. It is crucial to note that the household wealth index in the DHS survey serves as an indicator of household economic status rather than directly reflecting socioeconomic status. Clarifying this distinction will ensure the accurate interpretation of results related to socioeconomic disparities.

**Response**: We agree with the editor on this point. In fact, there are many proxy indicators for socioeconomic status. We have noted that the wealth index contained in the SADHS is essentially a measure of household economic status and that socioeconomic status is broader, but we’ve used the wealth index only as a proxy. Also, we have included this under the sudy’s limitations.

4. Consideration may be given to including marriage and employment as determinants in the analysis. If not included, please provide a rationale for their exclusion to strengthen the conceptual framework of the study.

**Response**: We thank you for this important suggestion to add these variables to our model.

Marital status and employment variables have been included in our model as recommended. Consequently, we had to re-run our model, with updated results reflecting the addition of these new variables.

5. The manuscript would benefit from improvements in English writing quality.

**Response**: The entire manuscript has been re-edited to improve the English writing quality.

6. Please ensure that the manuscript preparation strictly follows the submission guidelines outlined by the journal, available at <https://journals.plos.org/globalpublichealth/s/submission-guidelines>

**Response**: Thank you

Reviewer’s Responses to Questions

**Comments to the Author**

**Reviewer #1:** This submission is not new. The authors inadvertently selected the option for a ‘new submission’ instead of submitting a revised version. I am managing the original manuscript.

xxxxxxxxxxxxxxxx

**Response**: We apologise for any errors in submitting the manuscript. We addressed the editor’s comments before (re)submitting the paper.

**Reviewer #2:** This is a well written article. I just have few comments that will make the article clearer to the readers:

**Response**: Thank you for your comment regarding our paper. We shall address your specific comments below.

1. Concentration Index Adjustment: The concentration index (CI) is a widely utilised measure in socioeconomic inequality analysis. However, it is crucial to address whether the CI has been adjusted to account for any biases or variations that could affect the results. To ensure clarity, the authors should include a detailed statement in the methodology section, specifying if the CI was adjusted. If adjustments were made, the authors should explain the rationale and the methods used to adjust the CI. Conversely, if no adjustment was made, the authors should provide a clear explanation for this decision

**Response**: We understand this comment in two ways and shall comment on and address both. The first involves adjusting the concentration index for binary variables used in this paper to align its values within the theoretical bounds of -1 and +1. The second concerns standardising the concentration index for age/sex variations and accounting for key explanatory variables.

For the first part, we are guided by a more recent paper appearing in Health Economics (Ataguba JE. (2022). A short note revisiting the concentration index: Does the normalisation of the concentration index matter? Health Economics; 31(7):1506-12) showing the possible counterintuitive nature of the “normalisation” of the concentration index. Therefore, we retained the standard concentration index, which still provides sufficient information to ascertain the nature of the distribution. Regarding the second aspect, the decomposition analysis we employed in this paper offers a more robust way of showing factors that explain the concentration index. This decomposition method uses a regression-based approach to demonstrate the key variables that explain socioeconomic inequalities. Please see our addition to the manuscript under the “Analytical methods for estimating health inequality” section:

“It has been suggested to normalise the concentration index when the variable of interest is binary like “obesity” and “overweight” as used in this paper to ensure that the theoretical value of C remains in the [-1,1] range [46]. However, the approaches suggested in the literature [46, 47] for normalising and adjusting the concentration index may produce results counterintuitive for policy to reduce the burden of overweight and obesity among the most affected populations [48]. Therefore, this paper did not normalise the concentration index but presents the results for the standard concentration index [48]. Further, the concentration index may be standardised to account for age/sex variations or for key explanatory variables [34, 45]. This adjustment was not necessary in this paper as the decomposition analysis that follows using these variables is a detailed approach to assessing and explaining key factors contributing to the concentration index.”

2. The Oaxaca Decomposition Approach: The article currently presents the use of the Oaxaca decomposition approach, but the rationale behind its selection remains unclear. To enhance the readers’ comprehension, the authors should elaborate on why this specific method was chosen. They should provide a brief explanation of the Oaxaca decomposition approach, highlighting its relevance and advantages in the context of their study. This expanded explanation in the methodology section will offer a thorough understanding of the approach and its application in analyzing the data.

**Response**: This is an important comment. We have justified choosing the Oaxaca-type decomposition approach instead of the total differential approach (TDA) or the recently proposed method by Ataguba and colleagues. As highlighted in the manuscript, one of the major drawbacks of the TDA is that it only applies to small inequality changes. We considered this to be very limiting. Also, the recent method only focuses on changes between- and within-socioeconomic inequalities.

Please see the addition below to address the reviewer’s comments.

“Three decomposition approaches may be used, namely the Oaxaca-type method, the total differential approach [49] and a recent method proposed by Ataguba and colleagues [50] that focuses on inequalities between- and within-socioeconomic groups. The Oaxaca-type decomposition approach used in this paper decomposes the change in socioeconomic inequality in overweight and obesity over time (i.e., between two time periods denoted by subscripts t and t-1) into changes in the concentration index (C_kt and C_(kt-1)) and elasticities (η_kt and η_(kt-1)) of the factors explaining and contributing to changes in socioeconomic inequalities in overweight and obesity. For each contributing factor or variable, changes in the elasticities between two time periods are weighted by the concentration index and the changes in the concentration index by the elasticities, as shown in equation 4. One limitation of the Oaxaca-type method was the difficulty disentangling changes within elasticities [49], which the total differential approach (TDA) presents as an alternative. Because this paper was not intended to unpack changes within elasticities, which is of limited policy relevance, it uses the Oaxaca-type decomposition approach widely used in applied research. Further, the total differential approach is only applicable for small changes in inequality [49], which makes its application rather restrictive compared to the Oaxaca-type approach that is encompassing. Also, the Oaxaca-type decomposition has the added advantage of being easily understood. The recently proposed decomposition framework by Ataguba and colleagues [50] decomposes changes in socioeconomic inequalities into changes between- and within-socioeconomic group health inequalities. It does not show variables or factors that explain changes in socioeconomic inequalities, a major focus of this paper.”

**Reviewer #3:** The manuscript has strengths but some of the issues needs to be addressed

**Response**: Thank you for your comments on our paper. We shall now address the specific comments below.

The authors have well presented the Oaxaca Decomposition approach used for socioeconomic inequalities in overweight and obesity among women of child bearing age.
Some typos need to corrected in preliminary section (Competing interests) and Data availability (review the journal criteria- the data needs to be provided in a supplementary file)

**Response**: Thank you for pointing out the typos. We have re-edited the manuscript to address this concern.

Regarding the data availability, we have reviewed the statement, which reads “PLOS journals require authors to make all data necessary to replicate their study’s findings publicly available without restriction at the time of publication. When specific legal or ethical restrictions prohibit public sharing of a data set, authors must indicate how others may obtain access to the data.”

We want to note that the DHS has not permitted us to repost their data elsewhere. Moreover, these datasets can be assessed free of charge but with DHS granting your request. So, the best we did was to refer the reader to the data itself and let them proceed to download it after logging into the system once they are granted access. We believe that this is very much in line with the requirements of PLOS journals.

Please see our wording in the mains manuscript:

“Data were obtained from the nationally representative South Africa Demographic and Health Surveys (SADHS) of the years 1998 and 2016 which are publicly available at <https://dhsprogram.com/data/available-datasets.cfm>.”

Rationale set well for the study
Socioeconomic status was biggest contributor for both years
Disaggregated results on age groups could be presented as it has covered a large range of age group.

Race was major contributor to change in both parameters

**Response**: Thank you for the feedback on our paper.

Recommendation on abstract needs to be revised based on results, Please revise as you have presented in the conclusion of main text (ie. not for all socioeconomic backgrounds; recommend on specific actions that could be taken for addressing the inequality gap)
Methods: line 3 to 4 could be rewritten as; Data was obtained from the nationally representative South Africa Demographic and Health Surveys (SADHS) of the years 1998 and 2016 which are publicly available at

**Response**: These are important comments. Based on the suggestions, we have updated the abstract and the main manuscript (i.e., the methose section).

In section Decomposing changes in the concentration index inequalities over 1998 and 2006 has been quoted I hope this is a typo as authors are comparing with the years 1998 and 2016
There are observed differences in sample sizes quoted by authors in different sections (data sources and summary statistics) which needs to reconfirm and correction. If needed analysis should be re-conducted based on the sample size (I hope it is not the issue).

**Response**: The minor typo has been corrected. It should be 2016, not 2006, as the reviewer picked up. Thanks for picking that up. The sample size disparity has been rectified. We have included a short flowchart demonstrating the process used to arrive at the final sample size.

Analysis of WCBA residing in urban/rural areas vs education status could provide more precise relation on observed in-equalities. Do consider if possible.

**Response**: Thank you for this comment. We have included urban/rural sub-analysis as suggested by the reviewer. This was motivated by the significant impact of the area of residence on observed socioeconomic inequalities in obesity and overweight among WCBA. We want to note that the results from this re-analysis are very insightful and added value to our discussion.

Re-Formatting of tables according to journal criteria needs to be done

**Response**: Thank you for your comment. We have re-formatted the tables and hope they now meet the journal’s criteria.

Further discussion required for explaining possible reasons behind the differences observed with the current study and the findings of reference 33 who reported that obesity could be evenly spread between poorer and richer women which is contrasting in current study. Authors could further discuss on observed changes in these years which can have contributed to this difference.

**Response**: Thank you for raising this issue. Please note that this reference is now [34] in the revised manuscript.

We apologize for not conveying the message clearly and have now re-worded the texts. In fact, the concentration indices were positive for both men and women in that previous study [34]. It was higher for men (0.12) but lower for women (0.088). In both cases, the concentration of obesity was higher among adults from wealthier backgrounds than their less affluent counterparts. The notion of “even” was to demonstrate that it is more evenly spread among women irrespective of their socioeconomic background compared to men. In fact, the concentration index for obesity among women estimated from the previous study (0.09) was very similar to those estimated in the revised manuscript (0.058 for 1998 and 0.08 for 2016).

Therefore, we have edited the texts and have explained what makes our study different (value addition). We have highlighted that the results are not entirely different regarding policy conclusions except for the added analysis demonstrating the importance of key factors explaining changes in inequality between time periods. We believe that this addresses the reviewer’s concerns.

We thank the Reviewers and the Editor for helping us to revise and strengthen this manuscript. We hope that this revised version is now acceptable for publication in *PLOS Global Public Health* Journal.

Yours sincerely,

Mweete D. Nglazi, on behalf of the all authors
