# Supplementary material for: Did socioeconomic inequalities in overweight and obesity in South African women of childbearing age improve between 1998 and 2016? A decomposition analysis
Source: PLOS Glob Public Health. 2024 Nov 14;4(11):e0003719. doi: 10.1371/journal.pgph.0003719 (PMC11563443; doi:10.1371/journal.pgph.0003719)
Supplement: Attachment — Submitted filename: Response to reviewers.docx [file pgph.0003719.s008.docx]

PGPH-D-24-00977R1

Did socioeconomic inequalities in overweight and obesity in South African women of childbearing age improve between 1998 and 2016? A decomposition analysis

PLOS Global Public Health

Dear Editor,

We would like to thank the reviewers for their helpful comments to strengthen the above captioned manuscript. Please find below a point-by-point response to the reviewers comments.

Journal Requirements:

Please review your reference list to ensure that it is complete and correct. If you have cited papers that have been retracted, please include the rationale for doing so in the manuscript text, or remove these references and replace them with relevant current references. Any changes to the reference list should be mentioned in the rebuttal letter that accompanies your revised manuscript. If you need to cite a retracted article, indicate the article’s retracted status in the References list and also include a citation and full reference for the retraction notice.

Additional Editor Comments (if provided):

In the S1 Figure: Data cleaning flowchart, "Excluding pregnant women, women aged more than 49 years and observations without a complete set of covariates used for data analysis"
Please include the number of participants excluded for each of the following reasons in the followchart: pregnant women, women over the age of 49, and lacking a complete set of covariates used for data analysis.

**Response:** The S1 Figure; Data cleaning flowchart was revised as suggested

[Note: HTML markup is below. Please do not edit.]

Reviewers' comments:

Reviewer's Responses to Questions

**Comments to the Author**

1. If the authors have adequately addressed your comments raised in a previous round of review and you feel that this manuscript is now acceptable for publication, you may indicate that here to bypass the “Comments to the Author” section, enter your conflict of interest statement in the “Confidential to Editor” section, and submit your "Accept" recommendation.

Reviewer #3: All comments have been addressed

2. Does this manuscript meet PLOS Global Public Health’s [publication criteria](https://c05y1x9s.r.us-east-2.awstrack.me/L0/https:%2F%2Fjournals.plos.org%2Fglobalpublichealth%2Fs%2Fcriteria-for-publication/1/010f0192769ddd3b-1f790115-7963-4a10-bed9-537cf9589c62-000000/azfj4VY8Nfye8jaIdlL2_zh4AFA=179)? Is the manuscript technically sound, and do the data support the conclusions? The manuscript must describe methodologically and ethically rigorous research with conclusions that are appropriately drawn based on the data presented.

Reviewer #3: Yes

3. Has the statistical analysis been performed appropriately and rigorously?

Reviewer #3: Yes

4. Have the authors made all data underlying the findings in their manuscript fully available (please refer to the Data Availability Statement at the start of the manuscript PDF file)?

The [PLOS Data policy](https://c05y1x9s.r.us-east-2.awstrack.me/L0/https:%2F%2Fjournals.plos.org%2Fglobalpublichealth%2Fs%2Fdata-availability/1/010f0192769ddd3b-1f790115-7963-4a10-bed9-537cf9589c62-000000/C8_XXo0ZezccxF7jPmK4Fr9v-SM=179) requires authors to make all data underlying the findings described in their manuscript fully available without restriction, with rare exception. The data should be provided as part of the manuscript or its supporting information, or deposited to a public repository. For example, in addition to summary statistics, the data points behind means, medians and variance measures should be available. If there are restrictions on publicly sharing data—e.g. participant privacy or use of data from a third party—those must be specified.

Reviewer #3: Yes

5. Is the manuscript presented in an intelligible fashion and written in standard English?

PLOS Global Public Health does not copyedit accepted manuscripts, so the language in submitted articles must be clear, correct, and unambiguous. Any typographical or grammatical errors should be corrected at revision, so please note any specific errors here.

Reviewer #3: (No Response)

6. Review Comments to the Author

Please use the space provided to explain your answers to the questions above. You may also include additional comments for the author, including concerns about dual publication, research ethics, or publication ethics. (Please upload your review as an attachment if it exceeds 20,000 characters)

Reviewer #3: No further comments

7. PLOS authors have the option to publish the peer review history of their article ([what does this mean?](https://c05y1x9s.r.us-east-2.awstrack.me/L0/https:%2F%2Fjournals.plos.org%2Fglobalpublichealth%2Fs%2Feditorial-and-peer-review-process%23loc-peer-review-history/1/010f0192769ddd3b-1f790115-7963-4a10-bed9-537cf9589c62-000000/z46Pf6UIjiyFe_DDVsazwke8G_o=179)). If published, this will include your full peer review and any attached files.


**Do you want your identity to be public for this peer review?** If you choose “no”, your identity will remain anonymous but your review may still be made public.


For information about this choice, including consent withdrawal, please see our [Privacy Policy](https://c05y1x9s.r.us-east-2.awstrack.me/L0/https:%2F%2Fwww.plos.org%2Fprivacy-policy/1/010f0192769ddd3b-1f790115-7963-4a10-bed9-537cf9589c62-000000/jhMHspiQflv0uK5As0OaznLPHl8=179).

Reviewer #3: **Yes:** Rabindra Bhandari

**Response:** We are pleased that the reviewever has no further comments on the manuscript,

We thank the Reviewers and the Editor for helping us to revise and strengthen this manuscript. We hope that this revised version is now acceptable for publication in *PLOS Global Public Health* Journal.

Yours sincerely,

Mweete D. Nglazi, on behalf of the all authors
